# Public Knowledge, Attitudes, and Practices Regarding Brucellosis in Taif City, Saudi Arabia

**DOI:** 10.7759/cureus.40014

**Published:** 2023-06-05

**Authors:** Faisal K Al-homayani, Fai M Altalhi, Zohour A Almalki, Matooqa A Alnemari, Hanan H Alfaifi, Ghadi K Alsaadi

**Affiliations:** 1 Nephrology, Taif University, Taif, SAU; 2 Medicine and Surgery, Taif University, Taif, SAU

**Keywords:** taif city saudi arabia, practices, knowledge, zoonotic infection, brucellosis

## Abstract

Background: Brucellosis is a serious zoonotic infectious disease. Humans contract the disease by coming into contact with infected animals or their products. In Saudi Arabia, brucellosis is considered to be an endemic disease, with an annual incidence of 15.34 per 100,000 population from 2003 to 2018. Because of the devastating consequences for human health, raising awareness is an essential component in preventing brucellosis. Our study aims to assess the knowledge, awareness, and attitudes regarding brucellosis among the residents of Taif City, Saudi Arabia.

Methods: A descriptive, cross-sectional survey conducted in June-October 2022 targeted the population of Taif City, Saudi Arabia. The data were collected by an online questionnaire, which included questions on sociodemographic characteristics, awareness regarding brucellosis, behavior and attitude toward animals, and consuming animal-based products.

Results: A total of 743 participants were included. The participants were 18-70 years old, 63.4% were females, and 79.4% had a university education. Only 450 participants answered yes to the first question: “Do you know about brucellosis or have you heard about it?” Therefore, they were asked to answer knowledge questions. It was found that out of 450 participants, 46.9% demonstrated a “poor” knowledge level. Participants aged 26-55 years old demonstrated significantly more “good” knowledge than the other age groups (p = 0.001). Males demonstrated significantly more “good” knowledge (30.6%) than females (14.9%) (p < 0.001). The practices and attitudes of animal breeder participants (16.2%) were satisfactory because more than half of them did not participate in the birth of animals (53.4%), 50.7% did not participate in the birth with abortion, and approximately 61% used gloves when taking care of animals. The practices were unsatisfactory because 53.4% of the participants reported that they “always” eat the meat of animals they keep, and 64.4% reported that they personally slaughter sheep or cows from the herd.

Conclusion: Our study showed that most of the participants were aware of brucellosis; however, at the same time, the knowledge level regarding brucellosis was not satisfactory.

## Introduction

Owing to the serious effects on human health, brucellosis is a major zoonotic infectious disease of international significance, particularly in the Middle East. Humans contract the disease by coming into contact with infected animals or their products, primarily unpasteurized dairy products, including milk and undercooked meat. The disease is caused by several *Brucella* species, which are gram-negative bacteria, and cattle, sheep, goats, and camels are the primary vectors of this disease [1].

Brucellosis is a challenging disease to detect because of its vague nonspecific symptoms, including fever with a distinct pattern, night sweating, generalized bone pain and fatigue, arthralgia, arthritis, and lymphadenopathy, which may progress to involve multiple organ systems and cause serious complications, such as spondylitis, endocarditis with vegetation, interstitial pneumonia with hilar lymphadenopathy, neurobrucellosis, and organ abscesses [2,3]. Pediatric patients experience nearly the same vague symptoms as adults. Thus, every child from an endemic area who has a febrile illness and a history of consuming raw milk, dairy products, and/or animal contact should be evaluated for brucellosis [4]. Moreover, a recent Saudi systematic review showed that maternal brucellosis during pregnancy has been linked to an increased risk of spontaneous abortion, early birth, and intrauterine infection with fetal loss [5]. The accuracy of diagnostic inferences is affected by a number of factors, including a variety of false positives symptoms of human diseases, and a number of diagnostic issues.

In Saudi Arabia, brucellosis is still considered an endemic disease and has a higher incidence than the global average, with an annual incidence of 15.34 per 100,000 from 2003 to 2018 [6,7]. The higher incidence is frequently attributed to the religious and cultural beliefs of the region, because raising camels and livestock is an important component of the Kingdom’s heritage, and most people in rural regions frequently keep sheep and goats and regularly consume their meat and unpasteurized milk. All aforementioned reasons are considered to be risk factors for brucellosis. Therefore, the Saudi Ministry of Health (MoH) stipulates the need to periodically report brucellosis cases because it has been designated as a notifiable illness by the MoH [8].

Currently, the literature shows that despite a high burden of infection in many areas of the world, the World Health Organization (WHO) and the World Organization for Animal Health (WOAH) both regard brucellosis as a neglected zoonosis because it is not frequently given priority by healthcare systems [9]. A prior study done by Farhad Bahadori et al. in Iran, the second most brucellosis-endemic nation in the world, revealed that each year, 500,000 individuals are afflicted with brucellosis, and according to the WHO estimates, 45,000 of these individuals reside in the Eastern Mediterranean Region [10]. In certain regions of Asia and the Middle East, brucellosis is a significant health burden. Half of the nations with the highest burden of public health issues due to brucellosis are in the Middle East [10]. Numerous initiatives, including the deployment of control and eradication programs, have assisted many nations in reducing the frequency of brucellosis infections in both humans and animals.

Raising awareness is an essential and critical component in preventing any health problem to avoid its emergence. Therefore, our study aims to assess the knowledge, attitude, and practices among the Saudi population regarding brucellosis in Taif City, Saudi Arabia. Furthermore, we aim to assess the sources of information and their reliability, which will help to determine future action plans regarding brucellosis disease.

This article was previously presented as a poster at the 5th Annual KSUMSC Research Forum on March 3, 2023.

## Materials and methods

Study design

A descriptive, cross-sectional survey was conducted from June to October 2022 to assess the knowledge, attitudes, and practices regarding brucellosis among the population residing in Taif City, Saudi Arabia. Participation was voluntary, and informed consent was obtained from all participants. The institutional research ethics board approval was obtained from the Research Ethics Committee at Taif University (application number: 43-780) before performing any study procedure. Participants' identities were kept confidential.

Study participants

This study included males and females aged 18 years and older, who were residents of Taif City, and who agreed to participate in the study. Subjects who lived outside Taif City, aged less than 18 years, and who did not fill out the questionnaire were excluded.

Sample size

The sample size was estimated using the Qualtrics calculator (Qualtrics, Seattle, WA) with a confidence level of 95%, a sample size of 384, and a margin of error of 5%. The questionnaire was sent to 1000 simple and randomly chosen people, and we received 880 responses.

Data collection and instruments

A predesigned online self-administered electronic questionnaire in the Arabic language developed in Google Forms (Google, Mountain View, CA) was used to collect the data. The questionnaire contained three main parts. The first part included sociodemographic data (age, gender, nationality, marital status, and education level). The second part contained questions about knowledge regarding brucellosis causes, mode of transmission, clinical presentation, complications, prevention, and treatment. The third part focused on attitudes toward animals in terms of abortion, participation in animal birth, and exposure to excrement. Finally, the practices toward consuming homemade milk or cheese and their own animals' meat were evaluated [11]. The questionnaire was sent to the target sample by seven data collectors through social media apps (WhatsApp and Twitter).

Statistical analysis

After data were extracted, they were translated, revised, coded, entered into statistical software, and analyzed using SPSS version 23 (IBM Corp., Armonk, NY). Categorical variables were presented as frequencies and percentages. Continuous variables were expressed as mean and standard deviation. Pearson’s chi-square test was used to evaluate the statistical relationship between categorical variables. A P-value ≤ 0.05 was considered statistically significant.

## Results

Our survey received 880 responses, and we included 743 participants who satisfied the study's eligibility criteria. The socio-demographic analysis showed that 326 (43.9%) participants belonged to the 18-25 years old age group, 471 (63.4%) were females, 715 (96.2%) were Saudi nationals, 385 (51.8%) were single, 590 (79.4%) had a university education, and 682 (91.8%) were employed in non-health sector field (Table 1).

**Table 1:** Sociodemographic details of the participants

		N	%
Age (years)	18-25	326	43.9
	26-35	149	20.1
	36-45	140	18.8
	46-55	96	12.9
	>55	32	4.3
Gender	Female	471	63.4
	Male	272	36.6
Nationality	Saudi	715	96.2
	Non-Saudi	28	3.8
Marital status	Single	385	51.8
	Married	324	43.6
	Divorced	24	3.2
	Widowed	10	1.3
Educational level	Primary education	5	0.7
	Intermediate education	15	2.0
	High school education	130	17.5
	University education	590	79.4
	No primary education	3	0.4
Work in the health sector	No	682	91.8
	Yes	61	8.2

A total of 450 out of 743 (60.5%) participants reported that they have heard about brucellosis, and they were asked to answer knowledge questions. The most common sources of this awareness were relatives and friends (61.1%), social media (22%), and books (16.9%). Only those participants who had an awareness of the infection were asked to answer the 13 knowledge questions related to brucellosis. Approximately 73.8% of the participants knew that animals such as camels, cows, and sheep could get brucellosis, and 79.6% agreed that it causes infection in humans. Only 42.4% knew the reasons for the transmission of brucellosis, whereas 75.3% knew about the symptoms of the infection. However, only 32.4% correctly identified all complications of brucellosis. Approximately 59.6% knew that the infection can be transmitted from an infected animal to an uninfected animal, and the majority of the participants (83.3%) agreed that it can be transmitted from an infected animal to a human. However, only 39.1% agreed that it cannot be transmitted from an infected person to a healthy person. At the same time, only 32.4% knew that bacterial brucellosis causes serious illness up to death. The majority of the participants (89.8%) knew that brucellosis can be treated, and approximately 72.4% knew that bacterial brucellosis can be prevented by boiling the milk. More than half of the participants (54.4%) knew that there is a vaccination for animals against bacterial brucellosis. However, only a few participants (16.7%) knew that no vaccination is available for humans to prevent brucellosis (Table 2).

**Table 2:** Participants' responses related to knowledge items

		N	%
Which animals can get brucellosis?	Camels, cows, and sheep	332	73.8
	Poultry	7	1.6
	All mammals	70	15.6
	Don’t know	41	9.1
Can it cause infection in humans?	Yes	358	79.6
	No	53	11.8
	Don’t know	39	8.7
Brucellosis is transmitted by?	Eating uncooked food of infected animals	15	3.3
	Eating unpasteurized dairy products of infected animals	208	46.2
	Exposure to the secretions of infected animals	7	1.6
	All of the above	191	42.4
	Don’t know	29	6.4
What are the symptoms of brucellosis?	Arthritis	8	1.8
	High temperature	88	19.6
	All of the above	339	75.3
	Don’t know	15	3.3
What are the complications of brucellosis?	Meningitis	96	21.3
	Endocarditis	20	4.4
	All of the above	146	32.4
	Don’t know	188	41.8
Can the infection be transmitted from an infected animal to an uninfected animal?	No	37	8.2
	Yes	268	59.6
	Don’t know	145	32.2
Can the infection be transmitted from an infected animal to a human?	No	24	5.3
	Yes	375	83.3
	Don’t know	51	11.3
Can the infection be transmitted from an infected person to a healthy person?	No	176	39.1
	Yes	183	40.7
	Don’t know	91	20.2
Bacterial brucellosis causes serious illness up to death	No	94	20.9
	Yes	146	32.4
	Don’t know	210	46.7
Can brucellosis be treated?	No	8	1.8
	Yes	404	89.8
	Don’t know	38	8.4
Can brucellosis be killed when milk is boiled?	No	22	4.9
	Yes	326	72.4
	Don’t know	102	22.7
Is there a vaccination for animals against brucellosis?	No	24	5.3
	Yes	245	54.4
	Don’t know	181	40.2
Is there a vaccine (vaccination for humans to prevent brucellosis)?	No	75	16.7
	Yes	199	44.2
	Don’t know	176	39.1

**Figure 1:**
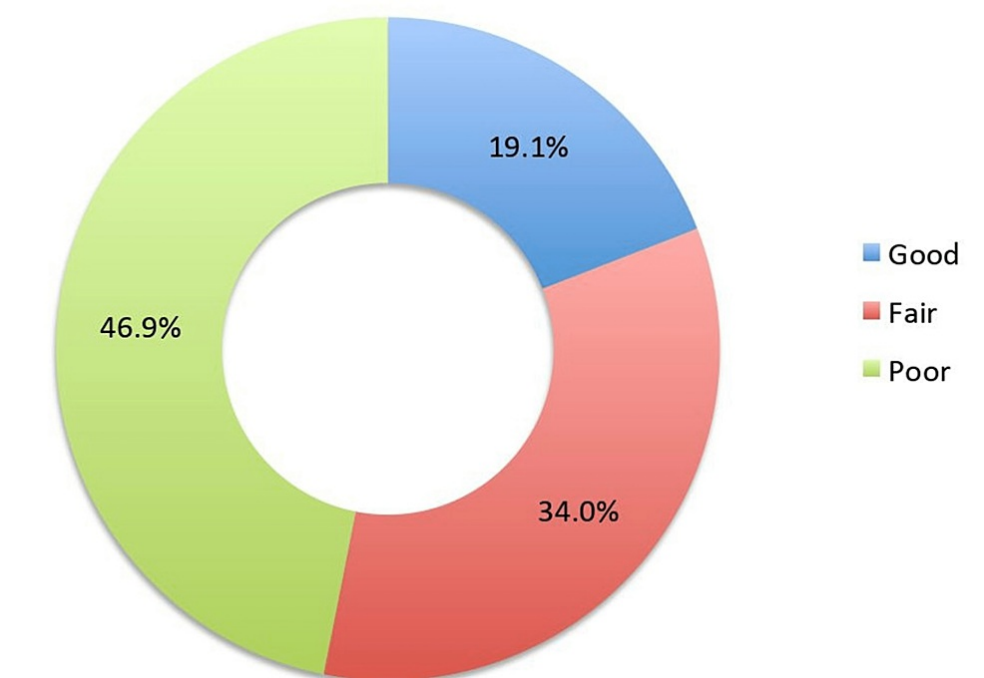
Knowledge levels regarding brucellosis (n = 450)

The total knowledge level of the participants was calculated based on the scores obtained for each knowledge item, where a correct response was given a score of "1" and wrong responses were given no scores. Then, the scores for each item were summed to obtain the total knowledge score, where the maximum score one could get was 13, and the minimum score was 0. The total scores were converted into percentages and then categorized as "good" (≥75%), "fair" (60-74.9%), and "poor" (<60%) knowledge levels. The performed analysis showed that 19.1% of the participants had a "good" knowledge level, whereas 46.9% demonstrated a "poor" knowledge level (Figure 1).

We evaluated the relationship between the knowledge level with different sociodemographic characteristics of the participants. When we compared the knowledge level with different age groups, it was found that the age groups of 26-35, 36-45, and 46-55 years demonstrated significantly more "good" knowledge than other age groups (p = 0.001). Males demonstrated significantly more "good" knowledge (30.6%) than females (14.9%) (p < 0.001). There were no statistically significant differences in knowledge levels in terms of participants' nationality, marital status, and education level (p > 0.05). However, the participants who worked in the health sector demonstrated significantly better knowledge (37.5%) compared to the non-health sector workers (16.95%) (p < 0.001). When we compared the knowledge levels between animal breeders and non-breeders, no statistically significant differences were observed (p = 0.710; Table 3).

**Table 3:** Knowledge level regarding brucellosis in terms of the sociodemographic characteristics of the participants (n = 450)

		Knowledge level			Total	P-value
		Good	Fair	Poor		
Age (years)	18-25	23 (16.0%)	42 (29.2%)	79 (54.9%)	144 (100.0%)	0.001
	26-35	12 (12.5%)	36 (37.5%)	48 (50%)	96 (100.0%)	
	36-45	27 (24.8%)	42 (38.5%)	40 (36.7%)	109 (100.0%)	
	46-55	23 (24.8%)	27 (35.5%)	26 (34.2%)	76 (100.0%)	
	>55	1 (4%)	6 (24%)	18 (72%)	25 (100.0%)	
Gender	Female	49 (14.9%)	112 (34%)	168 (51.1%)	329 (100.0%)	<0.001
	Male	37 (30.6%)	41 (33.9%)	43 (35.5%)	137 (100.0%)	
Nationality	Saudi	86 (19.6%)	150 (34.2%)	203 (46.2%)	439 (100.0%)	0.142
	Non-Saudi	0 (0.0%)	3 (27.3%)	8 (72.7%)	11 (100.0%)	
Marital status	Single	28 (15%)	57 (30.5%)	102 (54.5%)	187 (100.0%)	0.059
	Married	55 (23.1%)	89 (37.4%)	94 (39.5%)	238 (100.0%)	
	Divorced	2 (11.8%)	5 (29.4%)	10 (58.8%)	17 (100.0%)	
	Widowed	1 (12.5%)	2 (25.0%)	5 (62.5%)	8 (100.0%)	
Educational level	Primary education	0 (0.0%)	3 (60%)	2 (40%)	5 (100.0%)	0.528
	Intermediate education	1 (11.1%)	5 (55.6%)	3 (33.3%)	9 (100.0%)	
	High school education	10 (16.1%)	19 (30.6%)	33 (53.2%)	62 (100.0%)	
	University education	75 (20.1%)	126 (33.7%)	173 (46.3%)	374 (100.0%)	
Work in the health sector	No	68 (16.9%)	138 (34.3%)	196 (48.8%)	402 (100.0%)	0.002
	Yes	18 (37.5%)	15 (31.3%)	15 (31.3%)	48 (100.0%)	
Animal breeder	No	71 (18.8%)	126 (33.4%)	180 (47.7%)	377 (100.0%)	0.710
	Yes	15 (20.5%)	27 (37.0%)	31 (42.5%)	73 (100.0%)	

**Figure 2:**
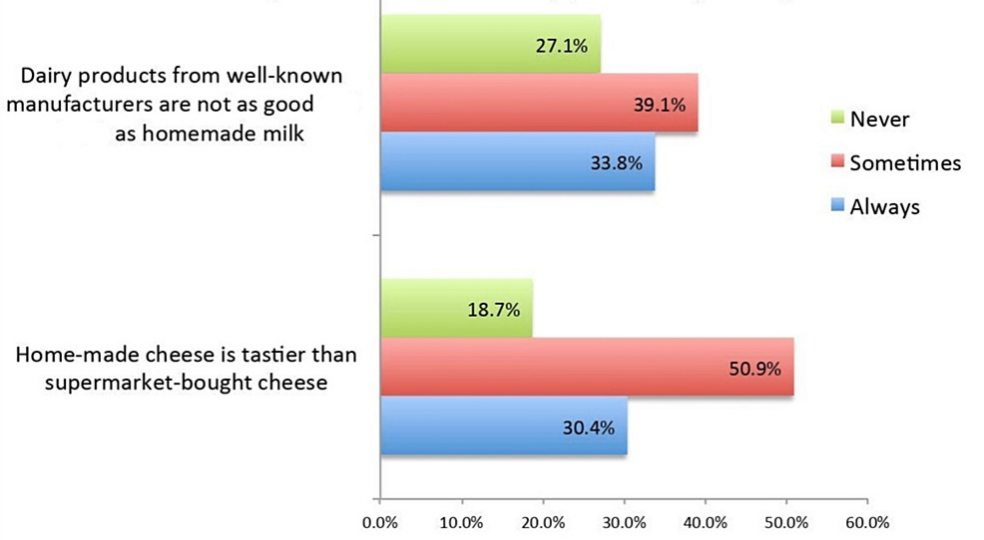
Perceptions related to dairy products (n = 450)

It was reported by 50.9% of the participants that homemade cheese is "sometimes" tastier than supermarket-bought cheese, whereas 18.7% did not agree with this statement. In addition, 39.1% believed that dairy products from well-known manufacturers are "sometimes" good compared to homemade milk, whereas 27.1% did not agree with this statement (Figure 2).

Out of 450 participants, only 73 (16.2%) reported being animal breeders; thus, we assessed the practices related to animal breeding among these participants. Approximately 53.4% reported that they "always" eat the meat of animals they keep, and 35.6% reported that they personally slaughter sheep or cows from the herd. Approximately 53.4% reported that they never participate in the birth of animals, and 38.4% mentioned that they do not use gloves when taking care of animals. More than half (50.7%) did not participate in the birth of a pregnant animal with an abortion. When we assessed the practices related to aborted animals, more than half (52.1%) reported that they bury the fetus, and 60.3% mentioned that they separate the aborted animals from the rest of the animals (Table 4).

**Table 4:** Practices related to animal breeding and burial of aborted animals (n = 73)

		N	%
Eat meat from the animals they keep	Always	39	53.4
	Sometimes	14	19.2
	Never	20	27.4
Personally slaughter sheep or cows from the herd	Always	26	35.6
	Sometimes	21	28.8
	Never	26	35.6
Participate in the birth of animals	Always	11	15.1
	Sometimes	23	31.5
	Never	39	53.4
Use gloves when taking care of animals	Always	26	35.6
	Sometimes	19	26.0
	Never	28	38.4
Participate in the birth of a pregnant animal with an abortion	Always	9	12.3
	Sometimes	27	37.0
	Never	37	50.7
What do you do with the aborted animal fetus?	Burial	38	52.1
	Burn	2	2.7
	Dog food	13	17.8
	Littering	20	27.4
What do you do with the animal after the abortion?	Keep it	23	31.5
	Kill	2	2.7
	Sell it	4	5.5
	Separate it from the rest	44	60.3

## Discussion

The findings of our study showed that knowledge about transmission, treatment, and hygienic measures related to brucellosis among our study population was not satisfactory, even though nearly two-thirds of the participants were aware of the disease. This means that although many participants were familiar with the term "brucellosis," their knowledge of the disease's zoonotic nature, mechanism of transmission, and symptoms in humans and animals trailed behind their awareness of the disease. Good awareness was observed for the type of animals that can get brucellosis and regarding transmission between humans. Male participants demonstrated better knowledge of transmission, treatment, and hygienic measures. This can be explained by the fact that men in Saudi communities tend to be the breadwinners and are more likely to work with animals and friends who may have some awareness.

Reduced efforts to manage and eventually eliminate brucellosis are hindered by a lack of education about the disease [12]. Given the zoonotic nature and public health relevance of brucellosis, the poor awareness and knowledge levels revealed in this study are of major concern. The public's health and the health of workers who are exposed to brucellosis on the job, as well as the safety of the public's food supply, require more attention because of the general lack of awareness and understanding of this disease. It has been noted that delaying medical assistance owing to a lack of understanding about the condition may delay both the diagnosis and treatment of the disease [13,14]. Complications from the condition may develop over time if therapy is delayed owing to a wrong diagnosis [15]. Furthermore, the poor brucellosis awareness and knowledge level of people participating in the livestock value chain may lead to negligence in disease prevention and inappropriate practices in handling, preparing, and preserving animal-based food, which poses a considerable threat to public food safety [16]. Individuals may be encouraged to take preventive steps against brucellosis infections if they are aware of behaviors that put them at risk such as consuming raw milk or undercooked meat and not wearing gloves when giving birth or handling abortion supplies.

Numerous variables are believed to influence the degree of brucellosis awareness and understanding. Multiple meta-analyses show that the education of the participants is correlated with higher levels of awareness and knowledge regarding brucellosis [17-26]. Awareness and knowledge of brucellosis are positively connected with prior experience and the prevalence of brucellosis in animals [27]. However, we did not observe significant differences in knowledge level based on the educational status of the participants. However, at the same time, the participants who worked in the health sector demonstrated significantly better knowledge than others. This is likely attributable to their medical training and experience gained over the course of their careers, highlighting the significance of education and training in raising brucellosis awareness among high-risk populations. According to research undertaken in southern Ethiopia, a lack of awareness-creating efforts by public health authorities and veterinary departments in the region may have contributed to a general lack of knowledge about zoonotic illnesses among the local population [28]. It has been claimed that a lack of knowledge among dairy farmers is caused by factors such as remoteness, a lack of health facilities, inadequate extension services, minimal training on the growing and handling of animals, a lack of health education programs, and low literacy rates [29]. A study conducted in the Aseer province of Saudi Arabia among parents reported that the majority of the participants (>90%) demonstrated good knowledge regarding brucellosis, and also the practices related to the disease were satisfactory [11]. The "One Health" concept currently promotes intersectoral and interdisciplinary collaboration in the management of zoonosis [30,31]. According to another study performed in Saudi Arabia, it was surprising that knowledge and practice on brucellosis and its prevention, diagnosis, and treatment were not satisfactory [32].

Improving knowledge and management of brucellosis and brucellosis-related information requires collaboration and communication across animal and human health sectors, agriculture, education, animal producers, and other relevant professional groups to gain support for a prevention campaign. In addition, health education about the condition among individuals at high risk is necessary. The importance of livestock breeders’ knowledge and awareness should be emphasized as a priority over other factors, such as the timing of vaccination, vaccine viability concerns, vaccine storage issues, quarantine issues, and the inability to predict the risk of brucellosis-related abortion in vaccinated animals.

Our research has some limitations. The participants' self-reported purchase and consumption of dairy products is a major limitation of this study. Nevertheless, this may be an under-reporting of the behavior, as consuming non-pasteurized dairy products is known to be a contributing factor for brucellosis. Additionally, we cannot rule out the possibility that there are unobserved differences between the municipalities that are skewing the findings. The cross-sectional approach of this survey does not allow us to understand the directionality of the relationship or causality; thus, longitudinal research may allow us to better comprehend the variables impacting behavior.

## Conclusions

In our study, most of the participants in our sample were aware of brucellosis; however, at the same time, the knowledge level of brucellosis was not satisfactory. To increase awareness of brucellosis, it is important to incorporate sufficient information about the cause and methods of transmission regarding brucellosis into public health education and awareness campaigns, particularly in remote regions.

## Appendices

Questionnaire

**Table 5:** Questionnaire items https://forms.gle/DzzFyEojRvFjLJ7t7

		N	%
Which animals can get brucellosis?	Camels, cows, and sheep	375	72.7
	Poultry	8	1.6
	All mammals	79	15.3
	Don't know	54	10.5
Brucellosis is transmitted by:	Eating immature food of infected animals	18	3.5
	Eating unpasteurized dairy products of infected animals	236	45.7
	Exposure to the secretions of infected animals	8	1.6
	All of the above	217	42.1
	Don't know	37	7.2
What are the symptoms of brucellosis?	Arthritis	11	2.1
	High temperature	99	19.2
	All of the above	386	74.8
	Don't know	20	3.9
What are the complications of brucellosis?	Meningitis	110	21.3
	Endocarditis	24	4.7
	All of the above	170	32.9
	Don't know	212	41.1
Can the infection be transmitted from an infected animal to an uninfected animal?	No	45	8.7
	Yes	305	59.1
	Don't know	166	32.2
Can the infection be transmitted from an infected animal to a human?	No	28	5.4
	Yes	423	82.0
	Don't know	65	12.6
Can the infection be transmitted from an infected person to a healthy person?	No	207	40.1
	Yes	202	39.1
	Don't know	107	20.7
Bacterial brucellosis causes serious illness up to death.	No	104	20.2
	Yes	174	33.7
	Don't know	238	46.1
Can brucellosis be treated?	No	12	2.3
	Yes	455	88.2
	Don’t know	49	9.5

Can brucellosis be killed when milk is boiled?	No	26	5.0
	Yes	372	72.1
	Don't know	118	22.9
Is there a vaccination for animals against brucellosis?	No	28	5.4
	Yes	288	55.8
	Don't know	200	38.8
Is there a vaccine (vaccination for humans to prevent brucellosis)?	No	89	17.2
	Yes	228	44.2
	Don't know	199	38.6
